# Footprints of the sun: memory of UV and light stress in plants

**DOI:** 10.3389/fpls.2014.00474

**Published:** 2014-09-16

**Authors:** Ralf Müller-Xing, Qian Xing, Justin Goodrich

**Affiliations:** ^1^Institute of Genetics, Heinrich-Heine-UniversityDüsseldorf, Germany; ^2^Institute for Molecular Plant Sciences, The University of EdinburghEdinburgh, UK

**Keywords:** abiotic plant stress, UV-B, epigenetic memory, stress signaling, *Arabidopsis*

## Abstract

Sunlight provides the necessary energy for plant growth via photosynthesis but high light and particular its integral ultraviolet (UV) part causes stress potentially leading to serious damage to DNA, proteins, and other cellular components. Plants show adaptation to environmental stresses, sometimes referred to as “plant memory.” There is growing evidence that plants memorize exposure to biotic or abiotic stresses through epigenetic mechanisms at the cellular level. UV target genes such as *CHALCONE SYNTHASE* (*CHS*) respond immediately to UV treatment and studies of the recently identified UV-B receptor UV RESISTANCE LOCUS 8 (UVR8) confirm the expedite nature of UV signaling. Considering these findings, an UV memory seems redundant. However, several lines of evidence suggest that plants may develop an epigenetic memory of UV and light stress, but in comparison to other abiotic stresses there has been relatively little investigation. Here we summarize the state of knowledge about acclimation and adaptation of plants to UV light and discuss the possibility of chromatin based epigenetic memory.

## ACCLIMATION AND ADAPTATION TO UV AND HIGH LIGHT

Plants use sunlight as an energy source and as an important environmental signal to regulate growth and development. Higher plants, such as the model plant *Arabidopsis thaliana* (*Arabidopsis*) use sunlight signals to regulate a whole range of developmental processes and adaptations including germination, de-etiolation, shade avoidance, stomatal development, circadian rhythm, and flowering. Beside regulation of growth and development, now termed “normal” photomorphogenesis, high light, and particular its integral ultraviolet (UV) part can induce stress responses in plants.

The type of stress response induced by light is determined by the fluence rate, exposure time, and whether plants have been acclimated by prior exposure to light. For example, light stress induced by UV-B includes DNA damage, production of reactive oxygen species (ROS), impairment of pathogen resistance and change of cellular processes (Jenkins, 2009; Li et al., 2013b). UV-B radiation promotes the generation of ROS as a result of metabolic disturbance and impairment of photosynthetic electron transport or due to increased activity of membrane localized NADPH-oxidases and peroxidases (Jenkins, 2009; Hideg et al., 2013).

Due to the formation of a stratospheric ozone layer that completely absorbs solar UV-C (<280 nm) and much of the UV-B, solar UV radiation now reaching the earth’s surface comprises only UV-A (315–400 nm) and part of the UV-B (280–315 nm; McKenzie et al., 2011). The potential of UV-B damage has increased in the last decades, which is caused by the depletion of ozone layer by release of chlorofluorocarbons (Moan, 2001). In the literature, plant UV-B responses are defined by low dose radiation (below or at ambient level, lower than 1 μmol m^-2^ s^-1^) or high dose radiation (above ambient level, 1–3 μmol m^-2^ s^-1^ or above) and short term (usually from seconds to hours) or long-term (usually from hours to days; Brown and Jenkins, 2008; Lang-Mladek et al., 2012; Hideg et al., 2013).

Low dose UV-B radiation can induce alterations in antioxidant status, e.g., regulation of glutathione pathways, phenylpropanoids, cinnamates, or flavonoids pathways, and pyridoxine biosynthesis pathways (Hideg et al., 2013). This mild and ecologically relevant UV-B radiation dose may trigger early adaptation, so that when conditions worsen, plants are protected from distress situations. In contrast, upon high dose UV-B radiation besides the same altered expression of genes involved in biosynthesis of phenols, the massive production of ROS in a short time over-rides the antioxidant capacity (Hideg et al., 2013), leading to a severe UV-B distress which can induce programmed cell death. In *Arabidopsis*, chronic low dose UV-B radiation can acclimate plants leading to changes in morphology and gene expression without causing stress symptoms (Hectors et al., 2007). UV-B induced morphological changes include decreased rosette diameter, reduced epidermal cell expansion, shortened inflorescence stem, and increased number of flowering stems (Hectors et al., 2007, 2010). Transcriptome profiling showed that UV-B induced morphogenesis was linked to phytohormone (auxins, brassinosteroids, and gibberellins) homeostasis and cell wall biogenesis (Hectors et al., 2007). Again, this low dose chronic UV-B induced morphogenesis is functionally uncoupled from stress responses by high UV-B at the gene expression level.

**FIGURE 1:**
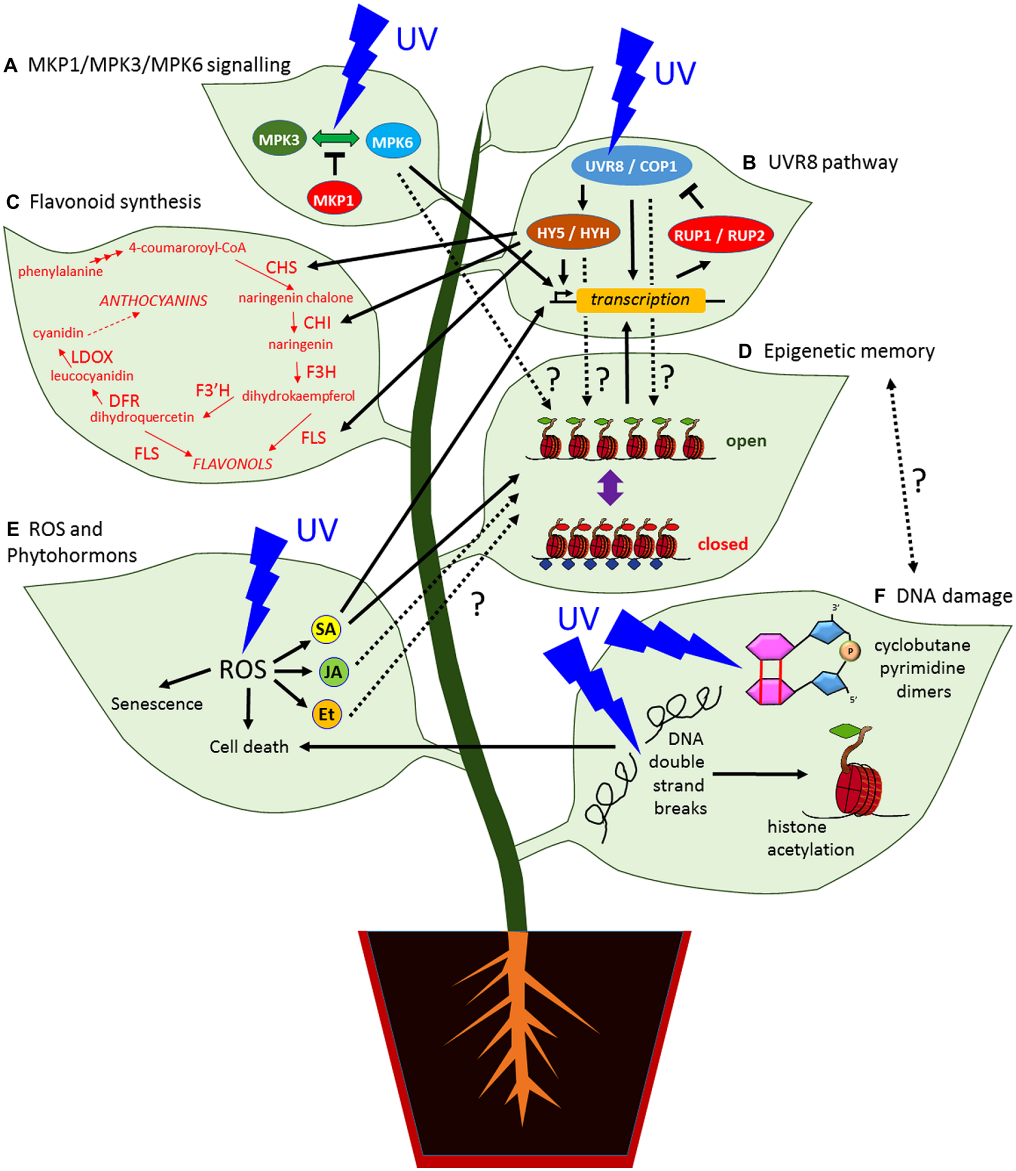
**Simplified model showing the UV radiation signaling pathways in plants and their possible links to an epigenetic based memory. (A)** The MAP kinase MPK3 and MPK6 signaling pathway can be activated by UV stress whereas MKP1 suppresses the activation. **(B)** UVR8 signaling pathway, the dimerization of the UV photoreceptor UVR8 with COP1 upregulates the expression of UV targets genes including HY5 and HYH which activate further UV response genes. In turn, the expression of RUP1 and RUP2 establish a negative feedback loop by direct protein binding. **(C)** HY5 can upregulate the expression of enzymes involved in flavonoid and anthocyanin biosynthesis. **(D)** Change of antagonistic epigenetic marks to light and UV stress could be part of an epigenetic memory but it remains unclear whether the activation of the MKP1/MPK3/MPK6 **(A)** or the UVR8 signaling pathway **(B)** by UV can establish persistent chromatin modifications. The phytohormone SA **(E)** can prime response genes by setting H3K4me3 at their loci that increase the transcriptional response **(B)** by a second stress. **(E)** UV-B promotes the accumulation of ROS that induces the production of phytohormones including SA, JA, and Et. High ROS concentration cause senescence and cell death. **(F)** UV radiation can cause cyclobutane pyrimidine dimers and DNA double strand breaks that can trigger cell death **(E)**. DNA damage repair pathways involve chromatin modifications including histone acetylation and remodeling to correct UV induced DNA lesions. There is a potential link between DNA repairs **(F)** and epigenetic memory **(D)**. CHI, CHALCONE ISOMERASE; CHS, CHALCONE SYNTHASE;; COP1, CONSTITUTIVELY PHOTOMORPHOGENIC 1; DFR, DIHYDROFLAVONOL 4-REDUCTASE; Et., ethylene; F3H, FLAVANONE 3-HYDROXYLASE; F3′H, FLAVONOID 3′-HYDROXYLASE; FLS, FLAVONOL SYNTHASE; HY5, ELONGATED HYPOCOTYL 5; HYH, HY5 HOMOLOG; JA, jasmonate; LDOX, LEUCOANTHOCYANIDIN DIOXYGENASE; MKP1, MAP KINASE PHOSPHATASE 1; MPK3 and MPK6, MITOGEN-ACTIVATED PROTEIN KINASES 3 and 6, ROS, reactive oxygen species; RUP1 and RUP2, REPRESSOR OF UV-B PHOTOMORPHOGENESIS 1 and 2; SA, salicylic acid; UV, ultraviolet radiation; UVR8, UV RESISTANCE LOCUS 8.

UV-B stimulates plants to accumulate specific flavonol glycosides which are produced in the vacuoles of epidermal and subepidermal cells protecting plants from UV-B irradiation (Emiliani et al., 2013; Hectors et al., 2014). Consistent with this, *Arabidopsis* mutants *transparent testa-4*, -*5*, -*6* which have reduced leaf flavonoids are highly sensitive to UV-B radiation (Li et al., 1993). The flavonoid biosynthetic pathway is mainly regulated by a group of genes encoding biosynthetic enzymes, including *CHALCONE SYNTHASE* (*CHS*), *CHALCONE ISOMERASE* (*CHI*), *FLAVANONE 3-HYDROZYLASE* (*F3H*), *FLAVONOL SYNTHASE* (*FLS*), *DIHYDROFLAVONOL 4-REDUCTASE* (*DFR*), and *LEUCOANTHOCYANIDIN DIOZYGENASE* (*LDOX*; **Figure 1C**; Tilbrook et al., 2013).

The relationship between UV and crops is considered very important as it affects yield. Consequently, UV-B responses were also investigated in crop plants including maize (Casati and Walbot, 2004; Falcone Ferreyra et al., 2010; Casati et al., 2011a), barley (Kravets et al., 2012), cucumber (Shinkle et al., 2010), grapevine (Pontin et al., 2010; Martinez-Luscher et al., 2013), woad (Chen, 2009), and Populus (Jia et al., 2009). Almost all of these publications studied acclimation or adaptation of plants to UV but only a few addressed the possibility of an epigenetic basis of these phenomena.

## SIGNALING PATHWAYS OF UV AND HIGH LIGHT AND THEIR TRANSCRIPTIONAL RESPONSE

Light perception in *Arabidopsis* involves different photoreceptors which cover a large part of the visible light spectrum and are mostly represented by gene families: five red and far-red light absorbing phytochromes (phyA-E); two UV-A/blue light absorbing cryptochromes (cry1 and cry2); two phototropins (phot1 and phot2) and three members of the Zeitlupe family (ZTL, FKF1, and LKP2; Schäfer and Nagy, 2006); and the recently characterized UV-B receptor UV RESISTANCE LOCUS 8 (UVR8; Kliebenstein et al., 2002; Favory et al., 2009; Jenkins, 2009; Fankhauser and Ulm, 2011; Rizzini et al., 2011). With the identification of UVR8 as the long sought after UV-B photoreceptor in 2011 (Rizzini et al., 2011), our understanding of the nature of UV-B perception, signal transduction, and downstream responses has been vastly advanced.

UVR8 was originally identified through characterizing an *Arabidopsis* mutant hypersensitive to UV-B, *UV resistance locus 8* (Kliebenstein et al., 2002). *UVR8* encodes a 440 amino acids seven-bladed β-propeller protein (Christie et al., 2012). At the N-terminus, UVR8 contains a nuclear localization domain and at the C-terminus a protein interaction domain which interact with CONSTITUTIVELY PHOTOMORPHOGENIC 1 (COP1), REPRESSOR OF UV-B PHOTOMORPHOGENESIS (RUP)1 and RUP2 during UV-B signaling (see below; Kaiserli and Jenkins, 2007). A key feature of photoreceptors is the utilization of chromophores to absorb light: UVR8 contains a cluster of 14 intrinsic aromatic tryptophans, with tryptophan-285 as a critical residue in the center of the protein structure, as a chromophore for UV-B perception (Rizzini et al., 2011).

UV-B can induce rapidly (within minutes) nuclear localization of UVR8 even at low fluence rates, however the constitutive nuclear localization of UVR8 is not sufficient to activate UV-B target gene expression (Kaiserli and Jenkins, 2007), suggesting more complex regulation for example through additional UVR8 subcellular localizations or post-translational modifications. UVR8 is structurally similar in the seven-bladed β-propeller fold to human regulator of chromosome condensation 1 (RCC1), which interacts with histone and DNA components of the nucleosome, although UVR8 potentially has greater conformational flexibility than RCC1 (Kliebenstein et al., 2002; Rizzini et al., 2011). UVR8 has also been shown to interact with chromatin via binding of histones (Brown et al., 2005). Chromatin immunoprecipitation (ChIP) assays revealed that UVR8 can bind to the promoter region of its downstream target genes, such as basic leucine-zipper (bZIP) family transcription factor *ELONGATED HYPOCOTYL 5* (*HY5*) which is a key regulator of photomorphogenesis (**Figure 1B**; Brown et al., 2005).

Prior to UV-B radiation, UVR8 exists as an inactive homodimer. Upon UV-B absorption by tryptophan residues, UVR8 undergoes structural change and immediate monomerization. The active UVR8 monomers interact directly with COP1 to initiate the UV-B signaling pathway (Rizzini et al., 2011; Heijde and Ulm, 2013). COP1 functions as an E3 ubiquitin ligase targeting key regulators for ubiquitination and degradation. In darkness, COP1 represses photomorphogenesis by promoting degradation of the transcription factor HY5 which plays an important role in de-etiolation when plants adjust their growth from darkness to light. Under white light condition or lack of UV-B, the activity of COP1 in promoting target protein degradation is mostly inhibited by photoreceptors (Lau and Deng, 2012). However, in the UV-B signaling pathway the interaction of COP1 with UVR8 plays a positive role but it is unclear what the COP1 targets are here or whether the E3 ligase activity of COP1 is significant as, for example, the specific and UV-B dependent UVR8–COP1 interaction is stable and does not lead to enhanced degradation of UVR8 (Favory et al., 2009). After exposure to UV *HY5* is transcriptional upregulated in a UVR8 and COP1 dependent manner suggesting a central role of UVR8, COP1, and HY5 in UV-B signaling (Brown et al., 2005; Brown and Jenkins, 2008). Signaling pathways usually contain negative feedback loops to avoid continuous activation. The WD-40 family proteins REPRESSOR OF UV-B PHOTOMORPHOGENESIS 1 (RUP1) and RUP2 are such negative regulators interacting directly with UVR8 and inactivating UVR8 during UV-B signaling (Gruber et al., 2010). *RUP1* and *RUP2* are transcriptional activated by UV-B in a COP1-, UVR8-, and HY5-dependent manner, while *rup1 rup2* double mutants showed an enhanced response to UV-B and elevated UV-B tolerance after acclimation (Gruber et al., 2010). RUP1 and RUP2 also regulate the UVR8 dimer to monomer ratio and negatively regulate the UVR8–COP1 interaction (Heijde and Ulm, 2013), indicating an important mechanism to dampen UV-B induced responses. For further information about UVR8-mediated signal transduction see Heijde and Ulm (2013) and Tilbrook et al. (2013).

There is some evidence that UV-B signaling may also occur independently of UVR8. For example, different groups of UV-B response genes are still represented in *uvr8*, *cop1*, *hy5* mutants upon UV-B radiation (Brown et al., 2005; Brown and Jenkins, 2008; Lang-Mladek et al., 2012). Mitogen-activated protein kinase (MAPK) signaling cascades have been proposed to be involved in high dose UV-B stress independently of UVR8-mediated UV-B signaling (Ulm et al., 2002; Gonzalez Besteiro et al., 2011). *Arabidopsis* plants with loss of *MAP kinase phosphatase 1* (*mkp1*) function are hypersensitive to acute UV-B radiation, but without showing impairment to UV-B acclimation. The MKP1-interacting partners MPK3 and MPK6 are activated by UV-B stress and are hyperactivated in *mkp1* mutants, suggesting that MKP1/MPK3/MPK6-mediated stress signaling pathway is crucial to UV-B tolerance in plants (**Figure 1A**; Gonzalez Besteiro et al., 2011). Alternatively it may be that at high fluence rates UV-B radiation in a short time directly causes destruction of proteins, membrane lipids, and chloroplast pigments, inhibits photosynthesis and growth, causes DNA damage, and leads to senescence and cell death. DNA has been proposed as a UV-B target since the 90s and the molecular mechanism of DNA damage and repair under the UV light is linked to photoreceptor signaling and DNA modifications (see later sections; Jansen et al., 1998). Another type of high dose UV-B responses might be mediated by ROS which leads to production of phytohormones, e.g., jasmonate, ethylene, and salicylic acid (SA, see below; **Figure 1E**) and activates general defense response to UV-B (Brosché and Strid, 2003). Therefore, it is not surprising that high dose UV-B induced programmed cell death (see below) might be independent from UV-B photoreceptor UVR8, and share some cellular hallmarks and regulatory machineries with other biotic and abiotic stresses, such as pathogen induced hypersensitive response (HR), heat stress, and water deficit (Nawkar et al., 2013). In contrast, under low fluence rates and longer term UV-B radiation, ROS level is controlled through signaling mediated by UVR8, COP1, and HY5/HY5 HOMOLOG (HYH), which activates antioxidant response (Brown and Jenkins, 2008; Bandurska et al., 2013; Hideg et al., 2013). This type of low dose UV-B responses might affect plant morphogenesis, metabolism, acclimation, and potentially memory of stress.

UV-triggered transcriptional changes were investigated on whole genome wide scales in different plant species including *Arabidopsis* (Ulm et al., 2004; Brown et al., 2005; Brown and Jenkins, 2008) and the crops grapevine (Pontin et al., 2010) and maize (Casati and Walbot, 2004; Casati et al., 2011a,b). For *Arabidopsis*, the whole transcriptome analysis by Brown et al. (2005) showed that UVR8 regulates genes involved in protection against oxidative stress (e.g., glutathione peroxidase), photo-oxidative damages (e.g., early light-induced proteins, ELIPs), and a range of transcription factors, transporters, and proteases which have key roles in protecting plants against UV-B. UVR8 also regulates genes involved in DNA repair, e.g., type II CPD photolyase PHR1 (PHR1; Brown et al., 2005; Heijde and Ulm, 2013). Last but not least, UVR8 regulates, dependent on HY5, most of the flavonoid biosynthesis genes, e.g., CHS, CHI, and FLS1 (**Figures 1B,C**; Brown and Jenkins, 2008). Notably, the upregulation of *CHS* by UV light is one of the oldest and best investigated transcriptional response to UV in *Arabidopsis* (Christie and Jenkins, 1996; Fuglevand et al., 1996; Hartmann et al., 1998; Safrany et al., 2008; Favory et al., 2009). In contrast to UV-B, the induction of *CHS* by UV-A is UVR8 independent (Jenkins and Brown, 2007). Furthermore, the kinetics of UV-B mediated induction of *CHS* expression are much faster (induction time < 5 min) than the UV-A/blue light-mediated induction (>30 min; Jenkins et al., 2001). In view of the fast response of *CHS* to UV-B and the general fast response of the UVR8 pathway, the question arises whether there is any need for an epigenetic memory of UV.

## CHANGES OF EPIGENETIC MARKERS TO LIGHT AND STRESS

The term epigenetic has been used in different ways, but currently is most usually used to describe mitotically or meiotically heritable changes in gene activity that are not caused by changes in DNA sequence, and that often show non-Mendelian properties such as reversibility. Epigenetic effects are typically caused by changes in chromatin, for which a wide variety have been described, including DNA methylation (DNAme), incorporation of histone variants, and covalent modifications to histone residues (Chen et al., 2010b). Exchanges of non-allelic histone variants lead to changes in chromatin structure and dynamics, therefore playing an important role in regulating transcription, DNA damage repair, regulating cellular response to environmental stimuli as well as their own epigenetic inheritance (Deal and Henikoff, 2011; Volle and Dalal, 2014; Yelagandula et al., 2014). In plants, the variant of histone H3, H3.3 is associated with expressed genes and active histone modifications such as H3K4me3 marks and RNA polymerase II (RNA Pol II), whereas H3.1 is associated with repressive marks (Stroud et al., 2012). By DNAme, a methyl group is added to the fifth carbon of a cytosine DNA nucleotide (Bird, 2002). Plant DNAme mainly occurs in three patterns, the CpG methylation, CHG (H indicating for A, C, or T), and CHH (an asymmetrical site) methylations (Chen et al., 2010b). The majority of modifications on histone H3 are lysine acetylations (H3K_ac) and H3 methylations (H3K_me). Whereas H3K_ac is generally associated with transcription activation and DNAme with gene repression, the effect of H3K_me is dependent of the specific residue and the number of methyl groups (me, me2, or me3). Typically, H3K9me2, H3K9me3, and H3K27me3 downregulate target gene expression, while H3K4me1, H3K4me2, H3K4me3, H3K36me2, and H3K36me3 upregulate target gene expression (Benhamed et al., 2008; Zhou, 2009; Chen et al., 2010b). Despite this extensive catalog of chromatin modifications, the evidence that they are epigenetic in the sense of heritable, self-propagating marks is much more limited. Methylation of DNA in the symmetrical CG and CHG contexts is mitotically and meiotically heritable, largely due to the existence of proteins that recognize the hemi-methylated DNA resulting from semi-conservative DNA replication and recruit DNA methyltransferases (for recent review see Kim and Zilberman, 2014). Gene repression mediated by polycomb-group (PcG) proteins is also mitotically heritable but usually reset between generations, as best characterized for the vernalization response in *Arabidopsis* (see Müller and Goodrich, 2011 and Song et al., 2013 for recent review). PcG repression is mediated in part through H3K27me3 methylation, deposited by the evolutionarily conserved histone methyltransferase complex polycomb repressive complex 2 (PRC2). Although it is not yet well understood how PcG repression is inherited through cell division, the finding that the PRC2 itself binds H3K27me3 is suggestive, as this may recruit PRC2 to newly replicated chromatin and maintain H3K27me3 methylation (see Margueron and Reinberg, 2010, 2011 for more information). It is also noteworthy that both for DNA methylation and for H3K27me3 methylation, demethylation enzymes have been identified (see Zhu, 2009 and Lu et al., 2011, respectively), indicating that these epigenetic marks can be actively reset. For further information on plant chromatin modification and remodeling in response to stress see Luo et al. (2012) and Zhu et al. (2012). Here, we first review the studies documenting changes in chromatin that occur following UV stress. We then discuss to what extent these changes may be causal in providing a memory of stress.

Chinnusamy and Zhu (2009) have challenged the idea that histone modifications and histone variant incorporation are per se epigenetic for two reasons: first, because many histone modifications are closely correlated with changes in gene transcription, it is not surprising that stress-induced gene regulation is associated with changes in histone modifications; second, the common definition of epigenetics requires mitotic or meiotic heritability (Chinnusamy and Zhu, 2009) and that is often not demonstrated in plant stress studies using the term epigenetics. However, Bird (2007) proposed a broader definition of “epigenetics” with less emphasis on heritability in part because non-dividing neural cells can show epigenetic phenomena (Hong et al., 2005). Studying drought stress responses in leaf cells that have predominantly ceased division, Ding et al. (2012) define “transcriptional memory” to mean that a type of information persists after the plant has recovered from the initial stress and that the “memory” influences subsequent transcriptional responses to future stresses. Following the definition of Bird (2007) we will use for all memory phenomena based on chromatin modification the term “epigenetic” although we are aware that some scientists prefer to use the term more specifically for heritable changes only. Epigenetic stress memory can be divided in three classes (Chinnusamy and Zhu, 2009): (1) “short-term stress memory” (∼ “transcriptional memory”; Ding et al., 2012), non-heritable but persistent changes in histone variants, histone modification, and/or DNAme that influence future transcription responses; (2) “long-term stress memory,” mitotically heritable changes; and (3) “transgenerational stress memory,” mitotically and meiotically heritable changes. Nevertheless, the design of the majority of the here presented plant stress studies does not enable to separate short-term and long-term memory, because whole seedlings were used and therefore, a mix of older leaves with ceased cell division and younger, including newly produced, leaves with dividing cells.

The relationship between a selected group of histone modifications (H3K4me3, H3K9ac, H3K9me2, and H3K27me3) and the expression of light response genes under white light/dark, red, blue, and far red light conditions were analyzed in *Arabidopsis* seedlings (Guo et al., 2008). The results show that there are significant differences in H3K4me3, H3K9ac, H3K9me2, and H3K27me3 histone modifications at representative gene loci with/without light, and that dark/light transitions could change H3K9ac status, which is also affected by light quality and light quantities, suggesting that different photoreceptors might be involved in regulating histone modifications (Guo et al., 2008). Interestingly, key components of the photomorphogenesis and UV signaling pathways, DET1, COP1, and HY5 seem to have a role in regulating H3K9ac, as in *cop1-4*, *det1-1*, or *hy5* mutants the H3K9ac status in representative genes is correlated with their transcription (Guo et al., 2008), suggesting that histone modifications might have a broad impact on light induced responses. In addition, histone acetyltransferases (HATs) and histone deacetylases (HDACs) have been reported to play a role in integrating light signals to histone acetylation in turn regulating light response gene transcription (Bertrand et al., 2005; Benhamed et al., 2006; Guo et al., 2008).

However, the links between changes in chromatin modification that are correlated with epigenetic mechanism and expression of genes involved in UV-B induced photomorphogenesis is not well established. A hint could be that CULLIN4 (CUL4)-damaged DNA binding protein1 (DDB1) interacts with DDB1 binding WD40 (DWD) proteins to act as E3 ligases as part of, or in conjunction with a large COP1/SPA/DET/FUS protein complex (COP1 is an essential component of the UVR8 signaling, see above) regulating photomorphogenesis and flowering time (Chen et al., 2010a). CUL4-DDB1- has also been reported to interact with MULTICOPY SUPPRESSOR OF IRA (MSI) proteins which are an integral part of the PRC2 complex catalyzing H3K27me3 methylation (Dumbliauskas et al., 2011). Decreased CUL4 activity reduces H3K27me3 at PRC2 target genes involved in flowering and it seems that a DDB1/CUL4/MSI4 complex activates PRC2, although it is not yet clear whether the E3 ligase activity is relevant for PRC2 degradation or ubiquitination (Pazhouhandeh et al., 2011). Whether COP1 E3 Ligase complexes could have a role in UV-B mediated histone modifications remains to be investigated.

UV-B exposure of Norway spruce (*Picea abies*) seedlings decreased the level of DNAme in needles, reflected in methylation changes in CCGG sequences (Ohlsson et al., 2013). Maize seedlings exposed to UV-C and gamma-irradiations displayed changes in satellite DNAme and chromatin structure, showing some correlation between DNAme patterns, chromosome aberration, germination rate, and resistance as well as adaptation to UV-C exposure (Sokolova et al., 2013, 2014). In *Arabidopsis*, the accumulation of *EARLY LIGHT INDUCED PROTEIN 1* (*ELIP1*) transcripts and proteins increased almost linearly with increasing white light intensities (Heddad et al., 2006). Also expression of *ELIP1* increases after UV treatment in *Pinus radiata* (Valledor et al., 2012) and *Arabidopsis*. In *Arabidopsis* the induction of *ELIP1* only needs low UV intensity and is dependent on the UV-receptor UVR8 and HY5 (Brown and Jenkins, 2008). Furthermore, the promoter of *Arabidopsis ELIP1* showed a significant enrichment of diacetyl histone H3 (H3K9ac2 and H3K14ac2) following UV-B exposure (Cloix and Jenkins, 2008). Interestingly, in unstressed *Arabidopsis* seedlings, the loci of *CHS*, *FLS,* and *DFR* are targets of the repressive mark H3K27me3, which is set by PRC2, in contrast to other components of the flavonol/anthocyanin synthesis pathway including *CHI*, *F3H,* and *LDOX* (Zhang et al., 2007). Conversely, *Arabidopsis* plants with highly reduced levels of MSI1, a central component of PRC2, do not respond to UV-B but show resistance to drought stress (Alexandre et al., 2009). In *P. radiata*, expression levels of *MSI1* and *SHMS4* encoding *S*-adenylmethione synthetase 4 which has a role in DNAme are highly upregulated after UV radiation (Valledor et al., 2012).

Although whole genome profiles of epigenetic marks after UV or high light treatment have yet to be reported, data from profiling of other abiotic stress are available. Recently Sani et al. (2013) published the epigenomes of *Arabidopsis* seedlings after hyperosmotic stress. ChIP-seq data of four histone modifications H3K4me2, H3K4me3, H3K9me2, and H3K27me3 revealed that the stress treatment established a long-term somatic memory. Several targeted transcription factors showed depletion of the repressive mark H3K27me3 after hyperosmotic priming which was associated with altered transcriptional responsiveness to a second stress treatment. Sunshine is often linked with heat and drought stress especially in summer and some subtropical and tropical regions. In *Arabidopsis*, developmental responses to higher ambient temperature have been shown to be mediated by variant histone H2A.Z. As temperature increases H2A.Z nucleosome occupancy decreases resulting in constitutive warm temperature transcriptome, and H2A.Z confers distinct DNA-unwrapping properties on nucleosomes, indicating a direct mechanism for the perception of temperature through DNA accessibility (Kumar and Wigge, 2010). Several studies support the idea of a trainable epigenetic memory of drought stress in *Arabidopsis*. A subset of dehydration stress–response genes, termed trainable genes, display transcriptional stress memory demonstrated by an increase in the transcription rate relative to previously non-treated plants (Ding et al., 2012). During recovery (watered) states, trainable genes produce transcripts at pre-induced levels, but remain associated with unusually high levels of H3K4me3 and of the phosphorylated serine 5 form (Ser5P) of RNA Pol II (Ding et al., 2012) associated with transcription initiation or pausing (stalling; Levine, 2011; Nechaev and Adelman, 2011). In a similar study, histone H3K9ac was enriched by drought and was rapidly removed from dehydration stress–response genes by rehydration (Kim et al., 2012). In contrast, histone H3K4me3 was gradually decreased by rehydration but was maintained at low levels after rehydration (Kim et al., 2012). These findings suggest that H3K4me3 but not H3K9ac could function as an epigenetic mark that provides a stress memory (Ding et al., 2012; Kim et al., 2012). Several of the dehydration stress–response genes are targets of the repressive mark H3K27me3 that limits, rather than prevents, transcription of these genes when responding to dehydration stress (Liu et al., 2014). A genome-wide analysis of the H3K4me3 epigenome in response to dehydration stress revealed that the changes of H3K4me3 from responding genes correlate with increased or decreased transcripts levels (van Dijk et al., 2010). Since many histone modifications including H3K4me3 are correlated with changes in transcription, it is not clear that changes in gene expression are caused by H3K4me3 or vice versa. More study is needed to see whether changes of H3K4me3 by drought stress, observed in the genome-wide analysis (van Dijk et al., 2010), are causal or necessary for transcriptional memory as suggested in the studies of Ding et al. (2012) and Kim et al. (2012) for a smaller gene set. Nevertheless, these studies of hyperosmotic and drought stress memory support the idea that acclimation to environmental stress is based partially on chromatin modifications. However, further investigation will be necessary to ascertain to what extent similar epigenetic mechanisms are involved in UV response and adaption in plants. To date, the increase of H3K9ac (e.g., at the *CHS* locus) is the only known histone modification triggered by UV-B treatment (Schenke et al., 2014) but further investigation will certainly reveal other modifications.

## THE ROLE OF THE PLANT HORMONE SALICYLIC ACID IN UV LIGHT RESPONSE

After encountering biotic stress (e.g., infection by a pathogen), plants often memorize the previous challenge, i.e., they are primed, so that their defense genes react faster and stronger during secondary stress. Even if the infection is localized, plants often acquire systemic immunity to further infections in tissue distal from the infection site. This phenomenon, termed systemic acquired resistance (SAR), requires the accumulation of the phytohormone salicylic acid (SA). The application of SA itself is sufficient to trigger resistance to biotic and abiotic stress (Ryals et al., 1996; Hayat et al., 2010). However, the epigenetic basis for SAR is not well understood.

A breakthrough in the plant stress field occurred recently with the demonstration that treatment of distal leaves with the synthetic SA analogue acibenzolar *S*-methyl (BTH) induces histone modifications at the promoters of defense genes encoding WRKY transcription factors (**Figures 1D,E**; Jaskiewicz et al., 2011). These histone modifications (H3K4me3 and H3K9ac) are generally correlated with activated gene expression. The modifications were induced at the WRKY genes although the WRKY genes were not transcriptionally induced during the BTH treatment (“priming” treatment). Treatment with a secondary stress transcriptionally activated the primed WRKY genes to higher levels than controls that had not experienced BTH treatment. These results suggest that the stress memory of plants is based on epigenetic histone modifications (Jaskiewicz et al., 2011).

There is strong evidence from several studies that SA levels increase in plant tissue after UV treatment, in addition SA treatment reduces the damaging effects of UV-B radiation on plants. SA levels increase significantly in tomato plants exposed to UV-B and SA accumulates in seedlings, roots, and leaves of barley after UV-B treatment (Yalpani et al., 1994; Bandurska et al., 2012; Bandurska and Cieślak, 2013). However, those experiments should be interpreted cautiously, as many of them used UV doses (or wavelengths) not present in the terrestrial environment (Ballare, 2014). On the other hand, the exogenous application of SA reduced the damaging effect of UV-B radiation on plants by up-regulating the activity of antioxidant enzymes and CHS and accumulation of anthocyanin (Campos et al., 2003; Ervin et al., 2004; Mahdavian et al., 2008). After 3 days exposing time, UV-B radiation resulted in a sevenfold increase in SA levels in *Arabidopsis* seedlings (Surplus et al., 1998). By contrast, short UV-C treatment induced SA synthesis only weakly in *Arabidopsis* (approximately twofold increase 6 h after irradiation). Four days after UV-C irradiation, the SA levels gradually decreased to values slightly lower than before treatment. (Yao et al., 2011). The SA accumulation promoted by UV-C was abolished in *SA induction deficient* mutants (Nawrath and Métraux, 1999). Furthermore, UV-C radiation upregulates the transcription of the *SA induction deficient 2* gene coding for the SA biosynthetic isochorismate synthase 1 enzyme (Martinez et al., 2004).

Taken together, these studies suggest that SA could have the function of a short-term memory for UV-B (as well as for other biotic and abiotic stresses) that may transfer the information to a long-term memory by setting active epigenetic marks on defense genes. However, more studies are required because the experimental design of different studies are non-coherent and the UV treatments and SA concentrations used were often unnaturally intense.

## DNA DAMAGE BY UV

Exposure to UV causes DNA damage including double strand breaks (Ries et al., 2000) and absorption of UV-B by DNA can induce the formation of covalent bonds between adjacent pyrimidines, giving rise to cyclobutane pyrimidine dimers (CPDs) which cannot be recognized by RNA and DNA polymerase resulting in blockage of gene transcription and DNA replication (**Figure 1F**; Jansen et al., 1998). Evolutionary conserved DNA repair pathways involve chromatin modifications and remodeling to correct various DNA lesions including UV-induced damage (Schmidt and Jackson, 2013), which might link UV and epigenetic memory.

Light-dependent photolyases reverse pyrimidine dimerization and restore the native form of DNA (Jansen et al., 1998). The *Arabidopsis uvr2-1* mutant which contains a lesion in the type II CPD photolyase PHR1 displays hypersensitivity to UV-B and loss of photorepair of CPDs (Landry et al., 1997), suggesting that a functional DNA repair system is crucial to maintain genome integrity and therefore enhance light induced stress tolerance. Elevated UV-B radiation strongly induces expression of photolyase *PHR1* and a putative marker for recombination repair *AtRAD1*, and is accompanied by increased frequency of somatic homologous DNA recombination in *Arabidopsis* (Ries et al., 2000). Interestingly, in the progeny of UV-B exposed *uvr2-1* plants, which lacks photoreactivation repair of CPDs, the frequency of homologous recombination was higher than the parent population, suggesting that the recombination process induced by UV-B might affect the genome stability of future generations (Ries et al., 2000).

The mismatch repair (MMR) system is known for its role in recognizing and correcting mispaired or unpaired DNA bases induced by UV radiation in bacteria, yeast, plants, and animals (Lario et al., 2011). MutS homolog (MSH) family proteins are involved in the repairing system, and recently it has been shown in human cells that H3K36me3 which is linked to actively transcribed genome regions facilitates DNA MMR by targeting the MMR machinery to chromatin during the cell cycle (Li et al., 2013a). *Arabidopsis MSH2* and *MSH6* (which are targets of E2F transcription factors involved in cell cycle regulation) and maize MSH family genes are up-regulated by UV-B, and are involved in repairing CPDs and in cell cycle regulation, suggesting that plant MSH family members are involved in a UV-B-induced DNA damage response pathway (Casati et al., 2006; Lario et al., 2011). However, the link between histone marks and plant MSH family during MMR is not known. Rice UV-damaged DNA binding protein homologs (UV-DDBs) have also been shown to specifically interact with E2F factors and are most abundant in proliferating tissues under UV radiation (Ishibashi et al., 2003).

A broad range of abiotic stresses, including temperature, drought, and UV-radiation, can cause transient modifications of chromatin structure which are associated with short term stress response, or longer stress memory which are associated with stable configurations of chromatin structure and even can be transmitted to subsequent generations (Zhu et al., 2012). The structure of chromatin can be remodeled in several ways, including DNAme, histone acetylation, histone methylation, and ATP-dependent chromatin remodeling (Luo et al., 2012; Zhu et al., 2012).

*DECREASE IN DNA METHYLATION1* (*DDM1*), encodes an ATP dependent SWI2/SNF2 chromatin remodeling factor that is required for normal patterns of genomic DNAme in *Arabidopsis*, and in the *ddm1* mutant most DNAme is lost, particularly at heterochromatin, and H3K9me2 is largely replaced by H3K4me2 (Gendrel et al., 2002). *DDM1* transcription is repressed by UV-B radiation, and *ddm1* mutants are more sensitive to UV-B radiation than the wild-type plants and constitutively express high levels of DNA repair enzymes, suggesting that DDM1 might be involved in UV-B induced DNA repair (Questa et al., 2013). Transcriptional profiling of high-altitude maize landraces was performed to screen different UV response ecotypes, and among the UV-B responsive transcripts several clusters of genes were implicated in chromatin remodeling, indicating that several chromatin factors could be implicated in UV-B tolerance or resistance (Casati et al., 2006). When maize *NFC102/NFC4* or *SDG102/SDG26*, which are putative chromatin remodeling or histone modification genes, were knocked down by RNAi, the lines showed hypersensitivity to UV-B and displayed more DNA damage than wild-type plants (Campi et al., 2012). Because UV-B can induce histone H3 and H4 acetylation in maize (Casati et al., 2008), the effect of histone acetylation on DNA repair was also analyzed in *Arabidopsis*. When the role of the histone acetyltransferases HAM1 and HAM2 in DNA damage repair was investigated, the *ham1* mutant displayed more CPDs and increased DNA damage suggesting that at least HAM1 plays an important role in UV-B induced DNA repair in *Arabidopsis* (Campi et al., 2012).

ANTI-SILENCING FUNCTION1 (ASF1) is a key histone H3/H4 chaperone and interacts with histone acetyltransferases, which participates in a variety of DNA repair and cell cycle regulation processes by interacting with E2F transcription factors (Lario et al., 2013). The expression of *ASF1A* and *ASF1B* were induced by UV-B, and RNAi knockdown lines of both genes showed increased sensitivity to UV-B compared to wild-type plants, indicating that ASF1A and ASF1B are regulated by cell cycle progression and are involved in DNA repair after UV-B irradiation (Lario et al., 2013). To summarize, evidence shows that photolyase dependent photorepair, chromatin remodeling, and histone acetylation are important during DNA repair by UV-B, demonstrating that chromatin modifications are also involved in DNA repair in plants. Further studies will be necessary to clarify whether the changes in chromatin induced during DNA repair provide any memory of the origin of the DNA damage, UV (**Figures 1D,F**).

## IMPACT OF UV UPON TRANSPOSON ACTIVITY AND TRANS-GENERATIONAL MEMORY

Transgenerational memory is defined as meiotically heritable changes in organisms in response to experiences in the previous generations (D’Urso and Brickner, 2014). The silencing of transposable elements (TE) by DNAme is important for the stability of plant genomes and is one of the classical examples of how epigenetic information is passed through generations (Chandler and Walbot, 1986). UV-B radiation treatments can reactivate silenced *Mutator* (*Mu*) TE in maize (Walbot, 1992, 1999). After UV-B treatment, the expression of the two genes of the Mu*DR* TE is increased and is accompanied by an increase in histone H3 acetylation and by decreased DNA and H3K9me2 methylation, indicating that these chromatin changes may be causal in transposon reactivation by UV-B in maize (Questa et al., 2010).

Plant responses to abiotic stresses such as heat, drought, and radiation can increase the frequency of homologous recombination, and the offspring of parent plants previously exposed to these abiotic stresses display a similar increase in homologous recombination, higher DNA methylation as well as enhanced stress tolerance (Boyko et al., 2010). The molecular mechanisms underlining the transgenerational stress memory have remained poorly understood. One explanation could be that ROS, phytohormones, secondary metabolites are produced relatively early after stress, and then as the stress intensity increases a long lasting response including histone modifications and DNA methylations can be transmitted to progeny (Pastor et al., 2013). UV light has been applied to investigate transgenerational memory, as elevated UV radiation can cause DNA damage and genome instability, homologous recombination, chromatin remodeling, particularly DNA and histone modifications, which have the potential to be transferred to progenies. Molinier et al. (2006) have used UV-C and flagellin to mimic environmental stress and to investigate the heritable recombination rates triggered by stresses. They used transgenic reporter lines that allow for histochemical visualization of homologous recombination and they showed that homologous recombination increased even four generations after the exposure to stresses (Molinier et al., 2006). However, studies by other groups using similar UV-C stress conditions, showed that genes involved in somatic homologous recombination, such as *RAD51*, *BRCA1*, *MIM1,* and *ATM* were up-regulated in the stress treated parent generation but not in the subsequent two progeny generations, which was correlated with an increased somatic homologous recombination in the parent generation but not in the subsequent two progeny generations (Pecinka et al., 2009). UV-B treatment activated a transcriptionally silenced GUS reporter gene and also several stress-related genes and this was correlated with increased histone acetylation (H3K9ac1) in *Arabidopsis* (Lang-Mladek et al., 2010), which is consistent with previous reports on both maize (Casati et al., 2008) and *Arabidopsis* (Cloix and Jenkins, 2008). However, transmission of the UV-B stress effects on reporter gene activation was confined to a small number of cells in non-stressed progeny plants and did not persist beyond two non-stressed progeny generations; in addition inheritance of stress-induced release of gene silencing was antagonized by seed aging (Lang-Mladek et al., 2010).

UV-C, salt, temperature, and drought can induce changes in global DNA methylation and gene transcription but the effect was restricted to the subsequent generation (Boyko et al., 2010). The stress induced increase in somatic homologous recombination was impaired in *dicer-like2* (*dcl2*) and *3* (*dcl3*) mutants, and interestingly, the progeny of *dcl4* plants exposed to UV-C exhibited genome hypermethylation suggesting that Dicer dependent small RNA pathways are involved in transgenerational memory to environmental stress (Boyko et al., 2010). Beside the debate on transgenerational transmission of environmentally triggered epigenetic traits, the mechanisms of these effects are still not well understood. A recent paper has shown that decrease in DNA methylation1 (DDM1) and Morpheus’Molecule1 (MOM1), act redundantly to reset the stress induced epigenetic state to prestress state, and thus prevent the mitotic and meiotic inheritance of stress triggered epigenetic changes, which may eliminate the basis of a potential transgenerational memory in plants (Iwasaki and Paszkowski, 2014).

## CONCLUSION AND OUTLOOK

The life of plants differs fundamentally from animals inter alia in two key aspects; they are (i) sessile and (ii) missing a trainable nervous system. Nevertheless, plants monitor changes in their environment and are able to memorize and anticipate these changes. Numerous studies addressed acclimation and adaptation to UV and high light in the model plant *Arabidopsis* as well as in crop plants documenting the importance of the subject. Recently, the identification of the UV-B receptor UVR8 has advanced our understanding of the response of plants to UV and the analysis of the UVR8 signaling pathway showed rapid upregulation of UV response genes like *CHS* by UV. The fast response to UV radiation can even be observed in plants which have never been exposed to UV suggesting that memory in the meaning of an epigenetic context is not required and the observed acclimation to UV might rather be based on physiological changes, e.g., accumulation of umbrella pigments and thickening of the cell walls.

Here, we summarized several research findings which might indicate the existence of an epigenetic based UV memory in plants. We are aware that linking plant responses to UV light to epigenetic memory is to a large extent only speculation. Nevertheless, several UV target genes carry chromatin modifications which are often associated with epigenetic mechanisms. Studies that analyse changes in chromatin modification after UV treatment are still missing in a whole genome scope. Further, we have to consider that changes in chromatin modification triggered by UV may only reflect changes in “normal” transcription. Whether chromatin modifications by UV are rather an integral part of an epigenetic memory or are only part of the transcription control, further investigation of changes in the chromatin status after UV treatment will give us a better understanding of the UV response in plants. In addition, genetic or other interventions will be necessary to test whether chromatin modifications are causal for changes in transcription and stress memory.

## AUTHOR CONTRIBUTIONS

Ralf Müller-Xing, Qian Xing, and Justin Goodrich wrote the paper.

## Conflict of Interest Statement

The authors declare that the research was conducted in the absence of any commercial or financial relationships that could be construed as a potential conflict of interest.
